# The Nursing Supplement

**Published:** 1888-09-22

**Authors:** 


### The Nursing Mirror.

All Contributions for this Supplement should be addressed "The Nursing Mirror," care of The Editor, 38, Old Jewry, E.C.

### En Passant.

OUR LIST OF VACANCIES.—We have received a mildly indignant letter signed M. C. C., the writer of which protests that our list of vacancies is useless unless we give particulars of where to apply and before what date. Not long ago the writer was very anxious to obtain a certain post, but lost so much time in procuring the particulars we did not give that the appointment was filled before her application was sent in. We are sorry this was so, but to print a complete account of all vacancies every week would trespass largely on our space, and we have to consider the wishes of advertisers as well as of readers. We have sometimes had complaints from secretaries of institutions, that by giving prominence to their vacancies we have brought on them an undesirably large shower of applications.

THE ST. JOHN SISTERHOOD.—Miss Selina Wilson has undertaken to fulfil the duties of Superior to the community of St. John the Divine. The post is one of considerable responsibility and power, and only great skill in administration can enable the St. John nurses to maintain their present position. The Order supports four hospitals: a general hospital at Lewisham, a hospital for women and children at Poplar, St. Gabriel's for babies, and a lying-in home. Besides the sisters of the community there are over a hundred nurses who work in their service, and who are thoroughly well disciplined and trained. The care of such a large association must be a hard test for any woman, and we cannot do better than hope the late Sister Selina may follow in Miss Lloyd's footsteps, and be as successful as her predecessor.

THE WHITECHAPEL MURDER.—One of the nurses of the Whitechapel Union Infirmary had to give evidence in the inquest held on Annie Chapman, the victim of the last sensational murder; and is called by the *Globe* "an ignorant nurse" because she carried out orders received. It is not the least of the drawbacks to a nurse's life in a large hospital that she may often have to bear witness against those she has attended. It is far easier to most nurses to face ghastly sights than to face a crowded court of justice or a coroner's jury. The appearance of a nurse (if she be in uniform) in the witness box always causes an extra stir, and makes the ordeal still more trying. One point of the law must always seem strange to a nurse, and that is when she has nursed a would-be suicide back to life, and has to hand him over to the police to be tried for the attempt. A hospital nurse who has never been before a magistrate is lucky.

AN AMERICAN NURSE'S BOOK.—Miss Elizabeth Robinson Scovil has written a small book entitled "In the Sickroom; What to Do, How to Do It, and When to Do It for the Sick: the Art of Nursing." The author is a graduate of the Boston Training-school for Nurses in connexion with the Massachusetts General Hospital. In twenty chapters she makes clear what is most necessary to do in a sick-room. The little manual is intended for mothers, sisters, and friends who have not had the advantage of technical training, but by attention to these simple rules may make those dear to them very comfortable and hasten their convalescence. These sort of manuals are not nearly so common in the United States as here, and whereas most of our text-books on the subject are written by doctors, it seems as though in advanced America the nurses are going to write for themselves.

NATIONAL PENSION FUND FOR NURSES.—We publish this week an important letter from Mr. E. T. Clifford, the honorary manager of the National Pension Fund for Nurses. It is of the nature of a reply to some recent remarks by the *Lancet*. The managers of the fund ought to be deeply grateful to the *Lancet* for so persistently playing the part of shuttlecock to their battledore. The public are thereby innocently amused, and the Pension Fund is well advertised and furnished with opportunities of explaining in minute detail the general provisions of the published prospectus.

STRANGE DEATH OF A CLERGYMAN.—We are sure that many of our readers will be glad to learn that Mrs. Davis who had nursed the Rev. F. D. Eyre, and was supposed to have accelerated his death, was exonerated from blame by the jury, who found that it was the clergyman's wilful abstinence from food that hastened death, and that he was insane. Also many will gladly learn that we were right in presuming that Sir Maurice Fitzgerald had exaggerated his account of the quarrel about a Catholic nurse at the Valencia Cottage Hospital, and that the priest has not yet cursed from the altar those of his flock who have had their injuries attended to by the sister now in charge, who belongs to a Protestant Nursing Community.

STRANGE ADVERTISEMENTS.—The constant appearance of an advertisement for a nurse to attend to a "slightly mental gentleman" is most amusing. One would have thought such a gentleman would have required no nurse, but apparently the advertisement means a gentleman slightly lacking in ordinary mental power. Another advertisement which appeared lately for nurses had the following stipulation: "Persons of good voice and able to sing in the church choir indispensable." Perhaps nurses think that invalids are usually quiet; but there must be exceptions, for the following advertisement stares at you from a chemist's shop at Brighton:—"Sparkling Invalids' Invigorating Beverage."

COURAGE.—The remarks of our correspondent "M." in the "Mirror" for September 8th on the question of courage have called forth one or two sensible letters we are sorry we cannot print this week. The general opinion seems to prove that "M." overstepped the mark in saying that the nurse who has been revaccinated runs no risk of catching small-pox; she runs less risk than the nurse who has not been revaccinated, but that absolute immunity is secured by inoculation is disproved by the late outbreak of small-pox at St. Joseph's Industrial Schools, near Manchester. Here three of the sisters and five of the attendants contracted the disease. Two nurses state that they caught small-pox from their patients, though they had been revaccinated. On the other hand, "M." stated that it required more courage to nurse typhoid, typhus, and scarlet fever. With regard to the first, it is received in general hospitals in the ordinary medical wards, and if the nurse carries out the precautions taught her she ought never to contract it. If it was as infectious as small-pox it certainly would be banished to the fever hospitals; indeed some of our correspondents say it is not infectious at all, but only contagious. The courage necessary to nurse typhus is not often called for in this country; but if it were we are sure there would be many nurses who would calmly and cheerfully work through an epidemic of it (or of small pox), and never think what were the probabilities of falling ill themselves, for well they know "They never fail who die in a great cause."

### Original Articles.

### A SISTER'S HOLIDAY.—Part II.

The next morning I walked across to the infirmary directly after breakfast, and found Miss James already in the wards superintending the dressing of a hip case. The patient was a poor wee girl aged seven, and not only was the hip bone diseased, but also the pelvis, so that amputation was out of the case, and there was nothing to do but wait for the end. The child was not in a splint, but extension was applied by means of the old-fashioned apparatus, which consists of a pole sticking up from the foot of the bed at an acute angle, the pulley-wheel being far higher than the patient's head. Dr. Evans came in just as my eye was fixed on this extension, and immediately the matron introduced him, he said: "You were noticing that ridiculous apparatus?"

"Yes; we don't use these extensions much now in the South."

"No; they are wrong by all the laws of mechanics. At an angle like this it would require double weight to have the least effect; you must get new extensions next spring, when the alterations are done, matron."

"And for the weights, Miss James," I put in, "you ought to get Esmarch's shot cans; they are like milk cans, with graduated lines to mark each pound. Then, if you want a two pound extension, you fill the can with shot to the second line, and so on. They are much tidier and more convenient than weights."

We passed on to the next case, which was another little girl suffering from hip-disease, but not nearly so ill as the first. I was astonished that she was not in a Thomas's splint, for she had been in the infirmary some months. Dr. Evans, however, stated that Thomas's splints are not nearly in such great favour in the North as they are in London. A poor old woman with disease of the left elbow and shoulder-joint was dressed by Dr. Evans; here again amputation was no use, nor was the patient strong enough to bear an operation; it was another hopeless case. A drainage tube was inserted in the shoulder wound, and the whole dressed with salicylic wool bandaged on with gauze. In this infirmary as elsewhere the spray had gone out of fashion, and was never used for a dressing, and seldom for an operation.

In the men's surgical ward there was a case of gangrene, not very bad, a case of malignant tumour on the arm, which was to be operated on in the afternoon, and a small boy who had fallen from a ladder, and was brought in for concussion. His hair had been closely clipped by the matron, and he was sitting up in bed, looking very jolly. There was a lump over the left ear, and fluctuation was easily felt. Dr. Evans's opinion was that there was a green-stick fracture which would right itself in time. We all three tried to feel pulsation, but failed.

In the men's medical ward was a delightful old Highland drover, supposed to be suffering from dyspeptic headaches. He seemed rather hard of hearing, and I suggested that he had little English, but this he denied. Dr. Evans shook his head and asked a few questions. He then produced his watch, and holding it some inches from the patient's right ear, asked him could he hear it. The man heard no sound; the watch was held nearer and nearer till it touched the ear, but still he could not hear it. Dr. Evans turned to the matron and myself and remarked in a low tone, "This is not dyspepsia; this is disease of the internal ear." Then turning to the patient, he asked "When did you lose your foot"?

"More than thirty years sin', at Edinburgh. It was the professor himsel'—Syme, dinna ye ca' him?—who took it off."

"A Syme's amputation is it! Here, off with your boot and let us have a look."

The man took off the long leather sort of tube which reached from the knee downwards, and was studded with huge nails at the bottom.

"It is very heavy," I said. "Can you walk far in it?"

"Walk, es it? I walked here frae Edinburgh last year; aye me, but I've never been well sin' I left Scotland. That boot cost me ten shillings before I left, and it's wearing oot a'ready."

Meanwhile Dr. Evans was looking at the splendid stump which had been left by the well-known ankle amputation which still bears Syme's name. It was as hard as possible, but the mark of the flap well up on the front of the leg could easily be seen. It was strange to think the man could walk so well on it, for there was no calf to the leg; but, of course, those muscles would be useless where there is no foot.

There were several cases of phthisis in this ward, two of which were being successfully treated by inhalation of oil of the eucalypti. A small case which fitted over the nose and mouth was tied round the head; it had a small sponge between a sort of grating in front where the air entered, and the oil was dripped on this sponge. The smell of the eucalypti was very pleasant, and Dr. Evans said he had got splendid results from its use. The patient in the next bed was suffering, it was feared, from cancer of the stomach. He was troubled with constant sickness, and was wasted to a skeleton. He held up his stick of an arm for the doctor's inspection, and, mournfully shaking his head, remarked, "You'll be sending me home to the churchyard soon, sir."

In the women's medical ward there were two cases of eczema, from which I concluded that my northern friends did not regard that disease as contagious. One was a dear wee boy of two, who could say nothing but "Fine herrings," which he sang out as the men in charge of barrows of fish do. His hands were tied up in bags, to his great disgust. How trying is the irritation of eczema was shown by the next case, that of a woman whose legs were chiefly affected. The doctor, pointing to some marks, bade the nurse not put on so much bandage, nor so tightly.

"If I don't, sir, she slips her hands down under the dressing and scratches the places," was the reply.

When we had finished the round of the four large wards, Dr. Evans remarked that there was a new case in the small room downstairs, supposed to be infectious; did I desire to go or not? Of course I went, and was rewarded by hearing the whole case taken and written down. It is always interesting to hear a case taken, and not often possible to the sister in a London hospital. The unwilling answers dragged from the patient, the strange description of symptoms, the queer facts of the family history. In spite of many questions, much patience and careful sounding, Dr. Evans was not sure of his diagnosis of the supposed fever case, being rather inclined to believe it pneumonia. It was now twelve o'clock, and the morning round was over. Dr. Evans shook hands and went off to make up his medicines, and Miss James carried me off to the kitchen, where it was her duty to see the dinners dished.

When I returned to my friends for lunch, and in reply to questions, acknowledged I had spent the morning at the infirmary, I was greeted with showers of derision and sarcasm; but I was not to be squashed—"Yes!" I replied, "I know I am here for a holiday; that is, a time of pleasant rest and recreation, and I can get that best by pottering about the infirmary if they will let me. They have been most courteous and kind to be there, and are making this the nicest holiday I have ever enjoyed."

### A VOCATION FOR WOMEN.

"Of all the vocations which modern society leaves open to women, there is none so distinctively feminine as nursing. Its pursuit involves a daily cultivation and expression of the noblest and loveliest traits of character. The most bitter cynics, even whilst maligning women in almost all other departments of life, have still felt compelled to speak gently of her in the capacity of nurse."—*Dr. Gaspard Griswold.*

### Everybody's Opinion.

[*Correspondence on all subjects is invited. No communications can be entertained if the name and address of the correspondent is not given, or unless one side of the paper only be written on.*]

### THE QUESTION OF COURAGE.

IT is with much interest I have read in the "Mirror" of the 8th inst. "M's" remarks on the mistaken idea of the courage of a small-pox nurse. We run absolutely no risk, as we should not be allowed to enter a ward unless we had been recently revaccinated, and the work is not so hard as any other nursing. You certainly need to have quick ears, and be ever on the alert for the escapades of your delirious patients. Bad cases, too, are very loathsome to behold, but you run no personal risk; whereas in scarlet fever, diphtheria, etc., to a certain extent you do, though proper precaution will prevent a great deal in those cases. I think far more courageous is the woman who devotes herself to the care of the insane—she is ever in danger, which she is powerless to avoid; she may at any time receive a blow which will disable her for life. I believe it is only by experience that anyone could possibly form an idea of the grave anxiety and trials which fall to the lot of the asylum attendant, and they are, I fear, a class of women whose merits are very much under-rated by the community at large. Whilst other nurses are lauded on all sides by the public for their unselfish work, and so on, the existence of the asylum attendant appears to be forgotten. Happily, however, for them, their true worth is appreciated by those in authority over them, promotions are granted them strictly on their merits, and before the attendant is placed in charge of a ward she must have proved herself to be a trustworthy, capable, and courageous woman. T. A.

### NURSING AND EMOTION.

MAY I beg leave to add a few remarks to a "Letter from a Probationer" with only a short time of experience in hospital nursing? I was much surprised and grieved to read in her letter that her heart was already growing stony towards the poor sufferers under her care. I pity them very much, and beg to say she had better far seek some other employment. Let us remember, although the poor patients are laid by in a hospital, they need our care and love, and someone to comfort them in their sufferings. Nurses need sympathising hearts, and should be always ready to give a cheerful word when the patients say, "Good morning, Nurse." Let us at all times remember they are God's poor and needy ones. I trust there are many nurses who look upon their profession as something higher than just a means of earning their bread. "Whatsoever thy hand findeth to do, do it with thy might;" "not with eyeservice, as menpleasers: but as the servants of Christ, doing the will of God from the heart."

NURSE M. E. H.

[While we gladly print our correspondent's remarks, we beg to assure her that our Probationer did not become hard-hearted in the sense she fancies, but only found herself getting over that paralysing feeling of horror and pain at the sight of suffering, which hinders a nurse from being efficient and successful.—ED. *T. H.*]

### Appointments.

[It is requested that successful candidates will send a copy of their applications and testimonials, with date of election, to THE EDITOR, The Lodge, Porchester Square, W.]

LEICESTER INSTITUTION.—Miss Lydia Jane Tanner, now matron of the Sister Dora Convalescent Hospital, at Milford, has been appointed Lady Superintendent of Leicester Institution of Trained Nurses.

NEWARK HOSPITAL.—Miss Brancker, late of St. Bartholomew's, has been appointed matron of the Newark General Hospital.

MISS L. STEWART, of the Royal Free Hospital, Gray's Inn Road, W.C., has been appointed night superintendent to the Children's Hospital, Great Ormond Street.

### Anecdotes of Personal Adventure.

"TERRA COTTA'S" amusing article in last week's HOSPITAL will be read with interest, as thoroughly true to life, by those who know the poor. I have often wondered if other people come across the strange complaints to be met with in our country cottages. The hairy siplus, brown titus, and information of the lungs are very common, but information of the brain is surely a superior affection, while catanass in the eye and a pusey in the side (if a good Nonconformist, too!) are regarded by some of us as truly unique. At a neighbouring hospital "it took six doctors to look at" the leg of an old woman who had failed to derive benefit from attending a home-and-catholic inspensary, and these gentlemen "all said that such a balganical leg they never did see." Another patient was suffering from concussion of the liver and a plicit of diseases; and at the present time one of my poor friends, who claims to be of Irish abstraction, is taking medicine to inure her blood, and her sufferings at times "do make her that inheritable as you wouldn't never think." My case for this week is "creeping palaces," which sounds dangerous, but luckily the patient is not of this parish. Living on the confines of the county, our sick folk have the advantage of being able to "change the air" by simply crossing a bridge hard by; and this grand panacea has been earnestly recommended to new-comers. KEYRIA.

### Notices.

THE children's harvest festival at the Hospital for Sick Children, Great Ormond Street, will be held in the hospital chapel on Saturday, September 29th, at a quarter to five p.m.

### Vacancies.

The following vacancies are announced:—

**Nurses.**

Leicester Fever Hospital; £20.

Ipswich Nurses' Home; three, £22.

Kent Nursing Institution.

Canterbury Nurses' Institute; two.

Radcliffe Infirmary: two.

Home for Trained Nurses, Bath; two, £25.

Bradford Infirmary; (night) £25.

Victoria Home for Nurses, Bournemouth; several.

Throat Hospital, Golden Square.

Wrexham Infirmary; £25.

Kingston Home, Hull; several.

Truro Nurses' Home; two.

Sheffield General Infirmary; £21.

Hertford Infirmary; £25.

Northampton Nurses' Institute.

Sheffield Nurses' Home; several.

Rochdale Infirmary; £22.

Birkdale Trained Nurses' Home, East Southport; several.

Royal Surrey County Hospital, Guildford; (head night) £20 to £24.

**District Nurses.**

Exmouth. Penlan. Wilton.

**Probationer.**

Royal Hospital for Diseases of the Chest.

### Notes and Queries.

TO CORRESPONDENTS.—1. Questions may be written on post-cards. 2. Advertisements in disguise are inadmissible. 3. In answering a query please quote the number. 4. If a private answer is desired, a stamped addressed envelope must be forwarded.

**Queries.**

(44) *Nurse A.*—Having worn the "Squire" collars and cuffs almost since they became so fashionable when worn by Mrs. Kendal, I may say that I found none so fine and durable as those I got from Robinson and Cleaver's, Belfast, Ireland. The cuffs are 7*s.* 11*d.* per dozen, the collars 4*s.* 6*d.* Messrs. Robinson and Cleaver send, post free, any order over £1. —*Islands.*

*Nurse C.* asks if any of the readers of THE HOSPITAL can tell her where she can buy a book entitled "Diagnosis of Infectious Diseases," by Dr. Allan?

(62) Can you kindly inform me of any hospital where probationers are taken at the age of twenty? and oblige.—*C. O.*

**Answers.**

(61) *E. G. Martin* had better read the paper on "A Nurse's Progress" in our issue for August 11th. If a probationer enters for three years, at most hospitals she is paid £10 the first year, £15 the second year, and £20 the third year. If she only enters for a few months, she pays a guinea a-week.

